# Innovative Method of Stimulating Vegetative Propagation of Large Cranberry (*Vaccinium macrocarpon* Aiton) Using New Organic Initiators

**DOI:** 10.3390/ijms26136369

**Published:** 2025-07-02

**Authors:** Natalia Matłok, Małgorzata Szostek, Tomasz Piechowiak, Maciej Balawejder

**Affiliations:** 1 Department of Food and Agriculture Production Engineering, Faculty of Technology and Life Sciences, University of Rzeszow, St. Zelwerowicza 4, 35-601 Rzeszów, Poland; 2 Department of Soil Science Environmental Chemistry and Hydrology, Faculty of Technology and Life Sciences, Collegium of Natural Sciences, University of Rzeszów, St. Zelwerowicza 8b, 35-601 Rzeszów, Poland; mszostek@ur.edu.pl; 3 Department of Chemistry and Food Toxicology, Faculty of Technology and Life Sciences, University of Rzeszow, St. Ćwiklińskiej 1a, 35-601 Rzeszow, Poland; tpiechowiak@ur.edu.pl (T.P.); mbalawejder@ur.edu.pl (M.B.)

**Keywords:** *Vaccinium macrocarpon* Aiton, vegetative propagation, rooting, indole-3-acetic acid (IAA), naphthaleneacetic acid (NAA), indole-3-butyric acid (IBA), reactive oxygen species (ROS), catalase (CAT), superoxide dismutase (SOD), photosynthetic efficiency

## Abstract

Large-fruited cranberry (*Vaccinium macrocarpon* Aiton) is a species known for its highly valued fruit and is typically propagated vegetatively through the rooting of stem cuttings. Studies on the rooting of stem cuttings of large-fruited cranberry have shown that the morphological traits of the root system are a key indicator of the effectiveness of this process. To support rooting, gel coatings based on polysaccharides and containing auxins, especially the indole-3-butyric acid (IBA) W4 variant, were developed and applied. These significantly influenced root length (increase of 44.6% compared to control W0), surface area (increase of 32.4% compared to W0), volume (increase of 26.7% compared to W0), and average thickness, which translated into better nutrient uptake and a higher degree of plant nourishment. The W4 coating, combining mineral components, polysaccharides, and IBA, reduced transpiration and maintained moisture, promoting effective rooting. The associated metabolic changes were confirmed by analyses of oxidative stress markers and chlorophyll fluorescence. The study demonstrated that enhanced root system development was closely linked with the increased accumulation of macro- and micronutrients in the aerial parts of the plants, directly contributing to improved growth and potential yield. These findings highlight that effective rooting—achieved through the targeted metabolic stabilisation of the rooting environment—is essential for the successful vegetative propagation of large-fruited cranberry.

## 1. Introduction

American cranberry (*Vaccinium macrocarpon* Aiton) is a plant species belonging to the heath family (*Ericaceae*). This low-growing plant has small, narrow leaves and produces red, spherical berries. In its natural habitat, the plant’s stems range from 0.2 to 2 m in length [1]. The species occurs naturally but is primarily cultivated commercially due to its valuable fruit, which has wide applications in the food and pharmaceutical industries [2]. This species is characterised by some soil requirements and a high tolerance to adverse environmental conditions. *Vaccinium macrocarpon* Aiton is characterised by a preference for acidic, well-drained, yet moisture-retentive soils with a high organic matter content, typically peat or sandy substrates. It also exhibits a high tolerance to environmental stressors such as low temperatures, periodic flooding, and fluctuating water tables [3,4]. American cranberry plants can be propagated both sexually and asexually. However, seed propagation is rarely used in horticultural practice due to the high variability of offspring traits and the long period required to reach fruiting maturity. Therefore, this species is most commonly propagated asexually, which allows for the production of genetically identical plants, ensuring uniform crop characteristics and simplifying plantation management. This process is accomplished both through in vitro techniques and by propagating plants from stem cuttings [5,6]. American cranberry produces long, trailing shoots known as stolons, which are primarily used for vegetative propagation rather than sexual reproduction. These stems root at the nodes upon contact with the substrate, giving rise to new plants. This natural process enables the plant to rapidly colonise space and maintain a healthy population [7]. In cultivation, the vegetative propagation of American cranberry is used to establish commercial plantations for the production of these fruits. Healthy stolon stems are collected from the mother plants and planted in a suitably prepared substrate. Usually, stems are rooted in production pots, after which the rooted cuttings are planted in the field to establish plantations.

An alternative, less costly method involves evenly spreading freshly cut, unrooted plant segments (stems) across the planting area and pressing them down using a specialised device resembling a disc harrow. Young plants are thus planted in the field in late spring or early summer, and the newly planted stems require frequent watering (at least twice daily for several weeks) until they root [1,8]. However, this method carries a significant risk of rooting failure, which ultimately delays ground cover formation and reduces production profitability. To address this challenge, the present study introduced an innovative approach focused on the development of gel-based coating formulations with sustained-release properties, designed to enhance rooting efficiency under field conditions. These novel preparations, enriched with auxins, were applied to vegetative cranberry shoot segments before planting. Their effectiveness was evaluated through a field trial by assessing their impact on the root system development, nutrient uptake, and overall physiological status of the plants.

This study constitutes a notable advancement in the vegetative propagation of *Vaccinium macrocarpon* Aiton, offering a practical and effective approach to enhance rooting efficiency and improve plant establishment. The findings provide novel insights into the application of field-based stimulant technologies and emphasise the potential of gel-based coatings as a scalable and innovative solution within modern horticultural practices.

## 2. Results and Discussion

### 2.1. Morphological Traits of the Root System

**Figure 1:**
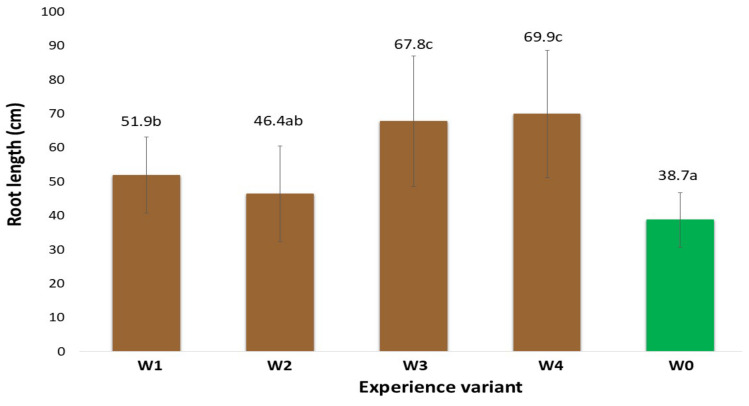
Total root length (in cm) of American cranberry plants depending on the treatment group and sampling date (mean ± SD). NOTE: Identical letters indicate no significant differences (*p* < 0.05) between respective treatment groups according to Tukey’s post hoc test (HSD).

**Figure 2:**
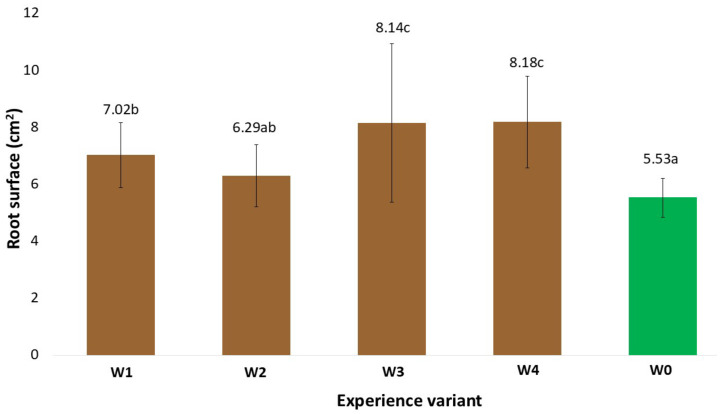
Total root surface area (in cm^2^) of American cranberry plants depending on the between respective treatment group and sampling date (mean ± SD). NOTE: Identical letters indicate no significant differences (*p* < 0.05) between respective treatment groups according to Tukey’s post hoc test (HSD).

**Figure 3:**
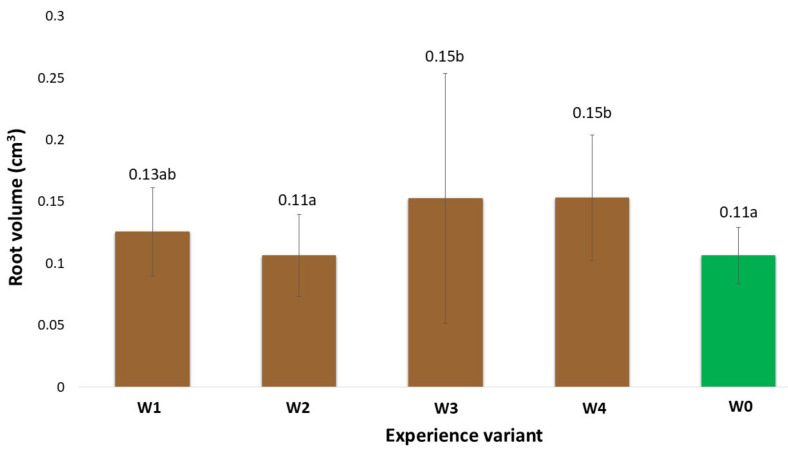
Total root volume (in cm^3^) of American cranberry plants depending on the between respective treatment group and sampling date (mean ± SD). NOTE: Identical letters indicate no significant differences (*p* < 0.05) between respective treatment groups according to Tukey’s post hoc test (HSD).

**Figure 4:**
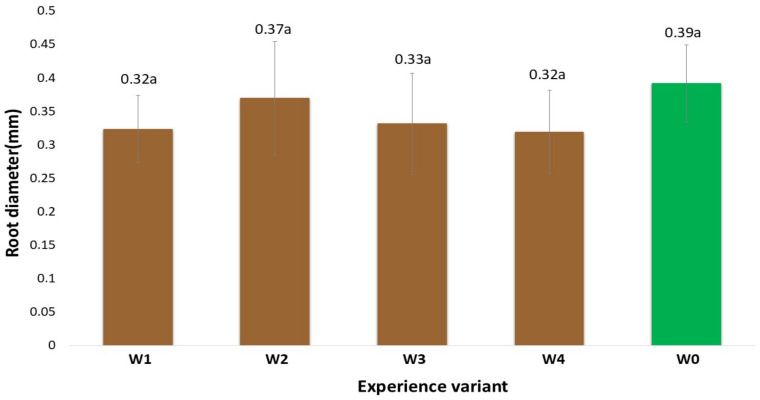
Average thickness of individual roots of American cranberry plants depending on the between respective treatment group and sampling date (mean ± SD). NOTE: Identical letters indicate no significant differences (*p* < 0.05) between respective treatment groups according to Tukey’s post hoc test (HSD).

**Figure 5:**
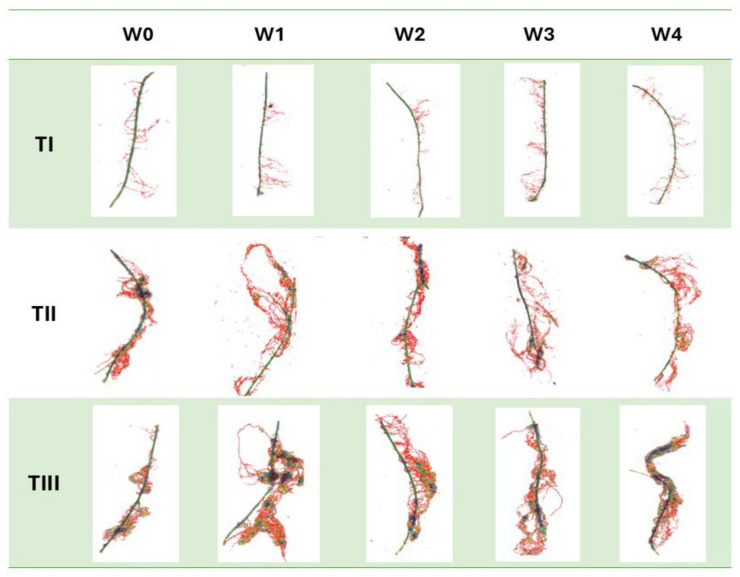
Root system morphology of American cranberry plants depending on the applied variant of the gel coating initiating root system development at selected time points (T1—30 days after planting; T2—45 days; T3—60 days).

The morphological measurements of the newly formed root system are reliable indicators of the effectiveness of the rooting process of the stem cuttings. The development of such a system enables the plants to uptake nutrients from the substrate, thereby intensifying their growth and development and, at later stages, yield [9]. The gel coatings used to initiate the rooting process of stem cuttings of American cranberry affected the total root length in varying ways (Figure 1). This variation depended on the specific coating preparation applied. In particular, the differences were due to the presence of auxin additives, which were the only variable component in the developed coatings. An analysis of the existing literature indicates that auxin content at a level of 1 mg dm^−3^ in preparations initiates root system formation in in vitro cultures [10]. The results obtained show that when supporting the rooting of stem cuttings under field conditions, cultivated on quartz substrates with a specific cultivation technology for American cranberry, the most effective coating (W4) was a composition of mineral components, polysaccharides, and other organic additives (glycine-betaine, Magno) supplemented with indole-3-butyric acid (IBA). The W4 coating resulted in an 80.6% increase in total root length compared to the control (W0, uncoated stems). Moreover, the total root surface area of cranberry plants treated with the W4 preparation increased by 47.9% and the total root volume by 36.5% in comparison to W0. Positive effects on the stimulation of new root system formation in large-fruited cranberry plants were also observed with coatings W1–W3; however, the measured root morphological parameters were generally significantly lower than those recorded for W4. This clearly indicates that this coating (W4) reduced transpiration of the rooted stems while maintaining appropriate moisture levels in their immediate vicinity, ultimately resulting in the formation of root hairs most effectively initiated by the addition of IBA auxins. IBA does not act directly as the main auxin signal but is converted in peroxisomes through peroxisomal β-oxidation into indole-3-acetic acid (IAA). The resulting IAA activates the auxin signalling pathway, leading to the expression of genes responsible for the initiation and development of root hairs [11]. It should be noted that the colloidal system of polysaccharides derived from agar in all analysed variants (W1–W4), besides retaining water, also preserved the mineral and organic components contained in the base coating. Similarly, the addition of the Magno preparation contributed to the supply of nutrients for the newly forming plants. It was also observed at the time of measurement that the applied auxins, particularly indole-3-butyric acid (IBA), had a positive effect. Various scientific studies clearly confirm the role of auxins, including IBA, as key regulators of plant growth in root development. Auxin biosynthesis, transport, and signalling play a crucial role in controlling root growth and development [11]. Additionally, the presence of auxins influenced the plants’ metabolic changes, which was confirmed by the activity levels of selected oxidative stress markers and chlorophyll fluorescence analysis. Similar results were observed in measurements of the total root surface area (Figure 2), total root volume (Figure 3), and average root diameter (Figure 4). The effects of the applied coatings on the root system of American cranberry plants at different time points after planting stem cuttings (T1—30 days after planting; T2—45 days; and T3—60 days) are presented in Figure 5. In horticultural practice, various rooting preparations are used to induce rooting from stem cuttings. Auxins of various types are documented active ingredients in such effective preparations [12]. Vegetative propagation strategies, besides powder preparations, include spraying cuttings with auxin-containing solutions, mainly NAA and IBA [13,14]. These solutions act in a similar manner to the developed and applied gel coatings that initiate rooting but do not maintain a constant concentration of the rooting agent near the plant tissues. However, the application and effectiveness of auxin sprays indicate that using auxin-containing solutions, particularly in the form of durable gel coatings, may enhance both the efficiency and the process of root system formation. In T3 the effect of the coatings becomes less distinct. However, it should be noted that only rooted stem cuttings were selected for the study. Nevertheless, the key period determining the effectiveness of planting is T1, where the most effective coating was W4 with added IBA.

### 2.2. Nutritional Status of American Cranberry Plants

A key factor determining plant growth and development is the nutritional status in terms of essential nutrients. The primary source of these nutrients for plants is through soil fertilisation, where nutrients are absorbed by plants through their root system. A well-developed root system is fundamental not only for early-stage growth but also throughout the entire vegetation period [15]. The applied coatings aimed to intensify the rooting process of stem cuttings of American cranberry, and a marker of the effectiveness of this process is the nutritional status of the plants, as measured by the content of macro- and microelements [16]. Among the macronutrients, nitrogen (N) plays a fundamental role as a building block of amino acids, proteins, nucleic acids, and chlorophyll, directly influencing biomass accumulation rates [17]. Our results show that the nitrogen content in the aboveground biomass varied depending on the coating type. Treatments W1 and W2 demonstrated a 9% higher nitrogen content than the control (W0), despite all coatings containing an identical amount of nitrogen. This finding supports the hypothesis that the extent and efficiency of the root system are the key determinants of nutrient uptake, as opposed to the nutrient availability in the immediate substrate. These findings are consistent with earlier work by Anbarasan and Srinivasan [18], who highlighted the synergistic relationship between root architecture, physiological activity (including root hairs and transport proteins), and microbial associations—particularly mycorrhizae—in promoting nutrient uptake and improving plant health. It has also been shown that root exudates and microbial communities in the rhizosphere modulate nutrient bioavailability [19] and that mycorrhizal symbioses can account for up to 80% of phosphorus uptake and significantly enhance nitrogen acquisition [20]. Interestingly, while the best nutrient uptake (especially nitrogen) was observed in W1 and W2, the most developed root systems were associated with treatments W3 and W4. This suggests that root function (e.g., root hair density, absorption surface, or microbial interactions), not just root mass or length, determines nutrient absorption efficiency. The importance of root system functionality at the early growth stage may outweigh the actual nitrogen concentration in the leaves or biomass. A strong initial root system can ensure consistent nutrient uptake throughout later growth phases. Jamaly et al. (2021) confirmed that NPK fertilisation directly affects cranberry plant development and yield potential, especially when combined with an already established root system [21]. Similar results were observed by Karami et al. [22], who demonstrated that the foliar application of polyamines (putrescine and spermidine) in Vaccinium species significantly increased the N, P, and K concentrations in leaves—up to 43%, 21%, and 18%, respectively. This illustrates that both root-related and systemic (foliar or hormonal) mechanisms can modulate nutrient accumulation, particularly when timed during critical growth stages. Moreover, nutrient absorption dynamics are governed not only by soil concentration but also by physiological processes such as active transport and osmotic gradients. Mass flow, diffusion, and root interception are the principal mechanisms of the nutrients’ movement to the roots. These mechanisms are all directly influenced by the morphology and physiology of the root system, further validating our findings. Karlsons et al. [23] demonstrated that the level of NPK fertilisation affects not only the growth and development of American cranberry plants but also shapes their yield potential. Their research was conducted on plants with fully developed root systems, confirming the correlation between the nutrient content in plant biomass and the size of the root system.

**Figure 6:**
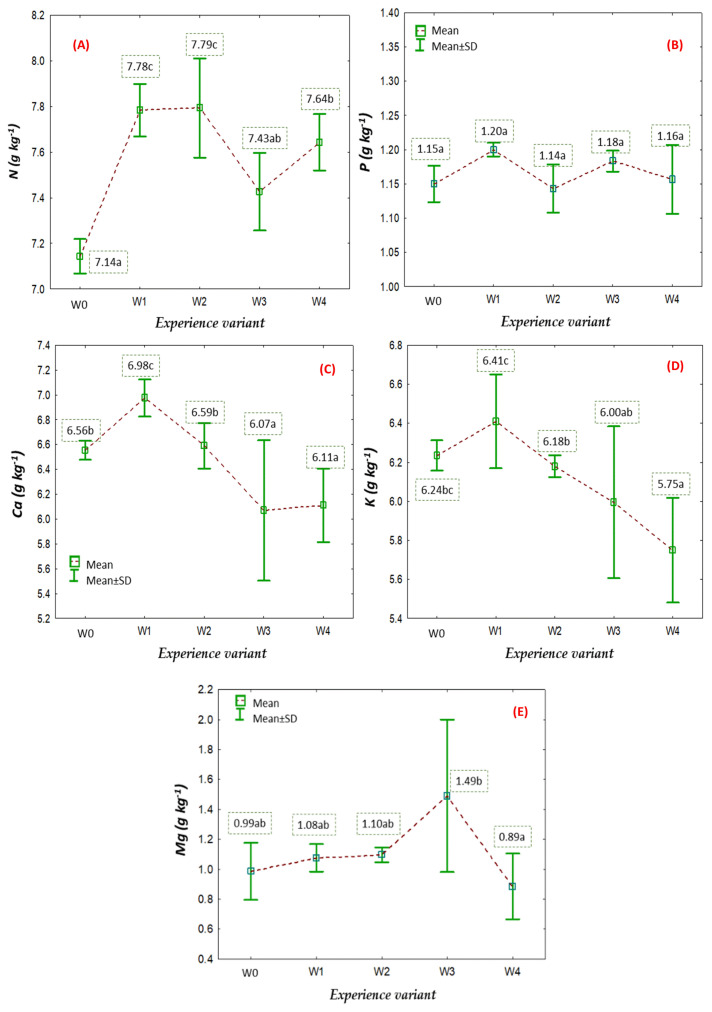
Content of N (**A**), P (**B**), K (**C**), Ca (**D**), and Mg (**E**) in the aboveground parts of American cranberry plants depending on the between respective treatment group (mean ± SD). NOTE: Identical letters indicate no significant differences (*p* < 0.05) between the respective between respective treatment groups according to Tukey’s post hoc test (HSD).

In cultivated plants, micronutrients are primarily supplied through foliar fertilisation treatments using preparations containing appropriate microelements (Figure 6) in assimilable forms [24]. However, the degree of their uptake depends on the initiation of root development, which directly influences the growth of aboveground biomass through which micronutrients are absorbed [25].

**Figure 7:**
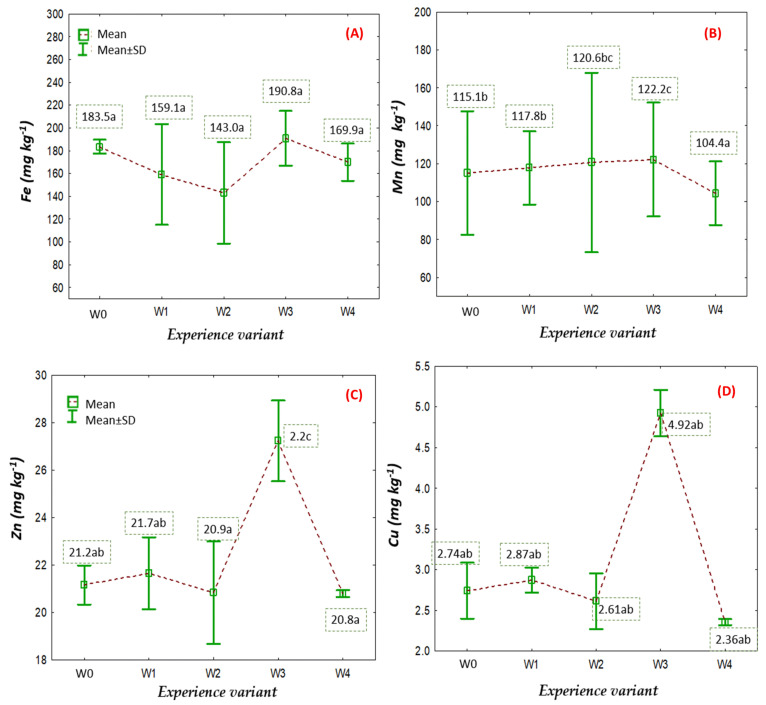
The content of Fe (**A**), Mn (**B**), Zn (**C**), and Cu (**D**) in the aboveground parts of large-fruited cranberry depending on the treatment group (mean ± SD). NOTE: Identical letters indicate no significant differences (*p* < 0.05) between the individual treatment groups according to Tukey’s post hoc test (HSD).

One of the important micronutrients in the cultivation of large-fruited cranberry is iron (Fe). The Fe content in the plants of this species, ranging from 40 to 80 mg kg^−1^ dry matter (d.m.), is considered optimal [26]. Meanwhile, the Fe content in the aboveground parts of plants, exceeding 80 mg kg^−1^ d.m., indicates a high level of this element. High Fe concentrations, ranging from 143.0 to 190.8 mg kg^−1^ d.m., were recorded in all respective treatment groups, and the observed differences were not statistically significant (Figure 7A). Such a high Fe content in the analysed aboveground biomass was most likely the result of a synergy between foliar fertilisation treatments and the high Fe content in the water used for irrigation. The average Fe concentration in the water was approximately 50 mg dm^3^.

An important micronutrient in plant cultivation is manganese. The biological functions of this element in plants are similar to those of magnesium. Both ions can substitute for or compete with each other in various reactions. Manganese activates about 35 different enzymes, most of which are involved in oxidation–reduction reactions. However, the most important physiological function of manganese is its participation in the photosynthesis process [27]. In this process, manganese, as a component of an enzyme associated with photosystem II, serves as an electron carrier in the photolysis of water (Hill reaction). Mn is also attributed a role in activating certain enzymes related to plant nitrogen metabolism and plays an important role in the biosynthesis of secondary products such as lignin, flavonoids, and indole-3-acetic acid [28]. For this reason, determining the nutritional status of plants in this element is crucial. It is assumed that manganese contents in the aboveground parts of large-fruited cranberry plants below 10 mg kg^−1^ indicate a deficiency of this element. The optimal range is considered to be broad, from 10 to 200 mg kg^−1^ [26]. The measured contents of this element (Figure 7B) analysed between the respective treatment groups were within the optimal range.

The zinc content (Figure 7C) was also determined in the analysed biomass of large-fruited cranberry plants. Except for the treatment group W3, the zinc content was similar, ranging from 20.8 to 21.7 mg kg^−1^ dry matter [26]. It should be noted that the recorded values fall within the optimum range for plants, which is 15 to 30 mg kg^−1^ dry matter.

Copper is an element that is part of many enzymes involved in numerous metabolic processes in plants, such as respiration, transformations of nitrogen compounds and sugars, and the lignification of cell walls. Copper is a component of plastocyanin found in chloroplasts, which functions as an electron carrier in the first phase of photosynthesis. Furthermore, this element participates in plant defence mechanisms [29]. The optimal concentration of copper in the leaves of large-fruited cranberry, indicating good nutritional status, ranges from 6 to 10 mg kg^−1^ dry matter. The recorded Cu contents (Figure 7D) in all analysed treatment groups were lower than recommended. This most likely resulted from the limited foliar fertilisation of copper in the plants.

### 2.3. Physiological Status Level of the Plants

Indicators of the physiological status of plants, including the intensity of metabolic processes, may include measurements of chlorophyll fluorescence parameters in leaves and the activity of selected enzymes in plant tissues [30].

**Figure 8:**
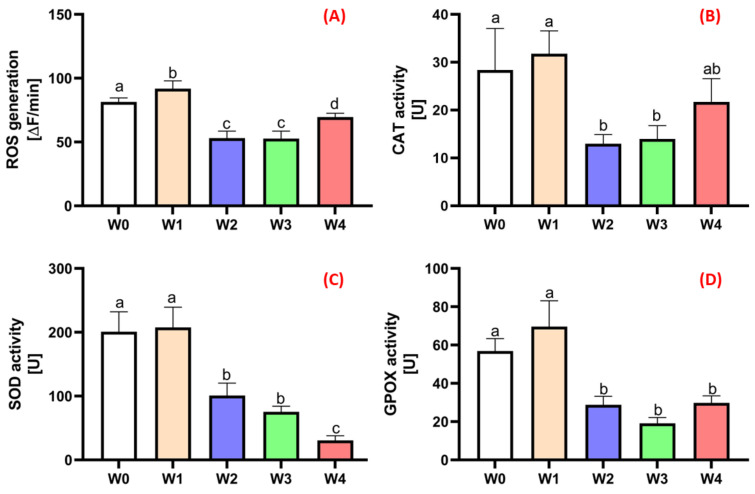
Results of the analysis of oxidative stress markers: ROS (**A**), CAT (**B**), SOD (**C**), and GPOX (**D**) in the roots of large-fruited cranberry. NOTE: Identical letters indicate no significant differences (*p* < 0.05) between the individual treatment groups according to Tukey’s post hoc test (HSD).

To determine the intensity of metabolism related to root system development by the plants, the activity levels of selected markers were measured. The level of reactive oxygen species (ROS) production, whose presence is associated with intensified mitochondrial activity, the main organelles responsible for energy transformations in cells, was assessed (Figure 8A). During intensive root system development, numerous cell divisions occur, resulting in the generation of ROS as a consequence of nutrient metabolism in the presence of oxygen absorbed from the environment. The highest level of ROS generation was recorded in roots taken from plants in the treatment group W1 (Figure 8A), where initiation was supported only by coating with a polysaccharide base. At the same time, plants from the W1 variant exhibited a significantly lower total root length (Figure 1), overall root surface area (Figure 2), and root volume (Figure 3) compared to the plants treated with coatings containing auxin additives, primarily IBA (W4) and NAA (W3). The addition of auxins maximised root system development while simultaneously limiting ROS generation, indicating a stabilisation of metabolic processes and minimisation of oxidative stress. The level of reactive oxygen species (ROS) plays a crucial role in the rooting process of plants, acting as a regulator of the signalling pathways involved in the initiation and development of adventitious roots. A moderate increase in the ROS concentration promotes the activation of genes associated with cell division and differentiation, thereby supporting the formation of meristematic tissue in the rooting zone. In contrast, the excessive accumulation of ROS leads to oxidative stress, damage to cell membranes, and the inhibition of physiological processes, ultimately reducing rooting efficiency. Excessive ROS presence in plant cells can be detrimental; therefore, physiologically healthy plant cells activate natural defence mechanisms [31,32]. Most commonly, superoxide anion radicals and hydrogen peroxide are generated in cells. To eliminate their excess, cells express enzymes, primarily catalase (CAT) (Figure 8B), superoxide dismutase (SOD) (Figure 8C), and guaiacol peroxidase (GPOX) (Figure 8D). The activity of these enzymes in the roots of large-fruited cranberry plants correlated with the level of ROS generation in plant cells in the different treatment groups. This indicates that plants undergoing intensive metabolic processes related to root system development strive for homeostasis.

**Figure 9:**
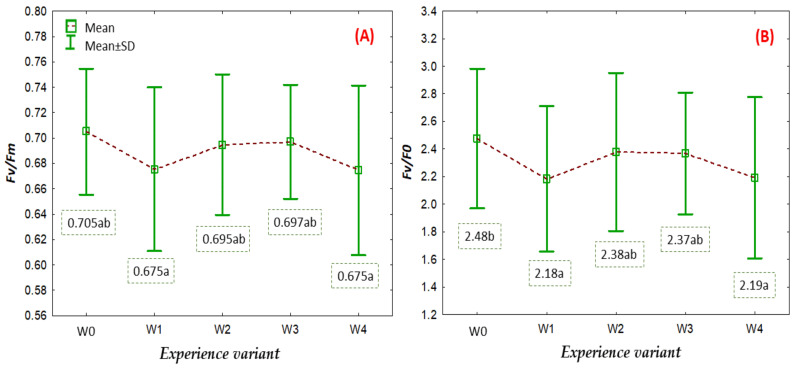
Maximum quantum efficiency of photosystem II (Fv/Fm) (mean ± SD) (**A**) and maximum quantum efficiency of primary photochemistry of PSII (Fv/Fo) (**B**) depending on the treatment group. NOTE: Identical letters indicate no significant differences (*p* < 0.05) between the treatment groups according to Tukey’s post hoc test (HSD).

A measure of oxidative stress in cells can be a non-invasive measurement of chlorophyll fluorescence parameters in the leaves of large-fruited cranberry plants (Figure 9). The Fv/Fm parameter (maximum efficiency of photosystem II) is the most popular comparative test used to detect stress in plants at its early stage [33]. An analysis of this parameter can reveal relationships between the physiological condition of the root system and the aboveground biomass of the plants. The recorded values of the parameter for all analysed variants were similar, in some cases being statistically different. Generally, the recorded values were lower than the optimal values, which range from 0.77 to 0.83 [34]. The observed values indicate that the aboveground parts of the plants were under stress. This condition resulted from the intensity of plant growth and development, mainly related to the formation of the root system.

## 3. Materials and Methods

### 3.1. Preparation of a Gel Coating for Inducing Plant Rooting

Gel coatings designed to initiate plant rooting were prepared, consisting of micro- and macroelements as well as selected auxins. For this purpose, the base solution (total volume of 2.5 dm^3^), containing ammonium nitrate (1650 mg dm^−3^), potassium nitrate (1900 mg dm^−3^), monopotassium phosphate (170 mg dm^−3^), magnesium sulphate heptahydrate (370 mg dm^−3^), calcium chloride dihydrate (440 mg dm^−3^), boric acid (6.2 mg dm^−3^), potassium iodide (0.83 mg dm^−3^), manganese(II) sulphate tetrahydrate (22.3 mg dm^−3^), zinc sulphate heptahydrate (8.6 mg dm^−3^), copper(II) sulphate pentahydrate (0.025 mg dm^−3^), cobalt(II) chloride hexahydrate (0.025 mg dm^−3^), iron(II) sulphate heptahydrate (27.8 mg dm^−3^), disodium EDTA (37.3 mg dm^−3^), sodium molybdate dihydrate (0.25 mg dm^−3^) (all form Merck KGaA, Darmstadt, Germany), LASTIM OSMO (Lallemand, Montreal, QC, Canada) (glycine-betaine > 96%, organic nitrogen 12%, 10 g dm^−3^), Magno (Darex, Działoszyn, Poland) (N—13.27%, K_2_O—6.9%, total amino acids—67%, peptides—4.3%, 10 g dm^−3^), agar (2.33 g dm^−3^), and water, was mixed using a Velp Scientifica magnetic stirrer (VELP Scientifica Srl, Usmate, Italy) and heated until a temperature of 95 °C was reached. Subsequently, the base gel mixture was divided into four 500 cm^3^ portions. To each portion, 0.5 mL of an ethanol solution of the appropriate auxins at a concentration of 1 mg cm^−3^ was pipetted (W2–W4). The solutions were mixed and cooled to room temperature.

### 3.2. Field Trial on Rooting of Stem Cuttings of American Cranberry Using Developed Rooting-Inducing Coatings

A controlled field trial on the rooting of stem cuttings of American cranberry was conducted using the developed rooting-inducing gel coatings on a quartz-based substrate. Vegetative segments of American cranberry plants of the ‘Pilgrim’ cultivar were obtained from three-year-old plants prior to the start of the growing season (25 April) by mechanically cutting stems at a height of 10 cm above the substrate. The collected plant segments were coated with the developed preparations using a 200 dm^3^ drum mixer (TOR INDUSTRIES, Gdańsk, Poland) at a weight ratio of 1:6. Applying these proportions resulted in a uniform coating of the preparation on the surface of the plant segments after 3 min of mixing. After 3 h of gelification, a gel coating formed on the surface of the coated stem cuttings, which were then planted into the substrate. The planted segments were irrigated to maintain a constant substrate moisture level at a 60% water holding capacity (WHC). During the growth and development period, the plants received standard crop protection treatments and fertilisation. 

### 3.3. Determination of Oxidative Stress Markers

Within 30 days of planting the stem cuttings, the rooted American cranberry plants were collected from all treatment groups for the determination of oxidative stress marker levels. The collected plants were cleaned of mineral substrate residues, and the underground parts were separated. These parts were then cut into segments approximately 10 cm in length using ceramic cutting elements to obtain analytical samples weighing 0.5 g each. The samples were homogenised with 4 cm^3^ of chilled 50 mM phosphate buffer (pH 7.4), containing a mixture of protease inhibitors and 0.05% Triton X-100 (Merck KGaA, Darmstadt, Germany). The homogenate was centrifuged using a Tabletop Centrifuge Megafuge-8 Series (Thermo Scientific, Waltham, MA, USA) at 10,000× *g* for 30 min at 4 °C, and the supernatant was used for the analyses of the generation of ROS and the activities of superoxide dismutase (SOD), catalase (CAT), and guaiacol peroxidase (GPOX) [34].

For ROS generation, 50 µL of the supernatant was mixed with 100 µL of phosphate buffer and 5 µL of 5 mM 2′,7′-dichlorodihydrofluorescein diacetate. The fluorescence increment was measured at 520 nm (emission) and 490 nm (excitation) using a Hitachi F7000 spectrofluorometer (Hitachi, Tokyo, Japan), and the results were expressed as an increase in fluorescence within 1 min by 1 g of the tissue [35].

The reaction mixture for determining superoxide dismutase (SOD) activity contained 5 µL of supernatant, 95 µL of 50 mM carbonate buffer (pH 10.2), and 5 µL of 10 mM epinephrine. The inhibition of epinephrine autooxidation was measured colorimetrically at 490 nm for 5 min using an EPOCH microplate reader (Biotek, Winooski, VT, USA). One unit of SOD activity was defined as the amount of protein required to inhibit the auto-oxidation of epinephrine by 50% [36].

Catalase (CAT) activity was assessed by measuring the residual hydrogen peroxide after the enzymatic reaction using ammonium metavanadate. Briefly, 10 µL of the supernatant was added to 80 µL of 10 mM phosphate buffer (pH 7.4) and 20 µL of 10 mM H_2_O_2_. After 10 min of incubation at 37 °C, 20 µL of 0.01 M ammonium metavanadate was added. Following 10 min incubation, the absorbance was measured at 425 nm. One unit of CAT activity was defined as the amount of enzyme that caused a decrease in absorbance of 0.01 per minute [37].

Guaiacol peroxidase (GPOX) activity was determined by mixing 20 µL of the supernatant with 80 µL of 50 mM citrate buffer (pH 5.0) and 20 µL of 8.26 mM guaiacol (in ethanol). The reaction was initiated by adding 20 µL of 8.8 mM H_2_O_2_. Changes in absorbance were measured kinetically at 470 nm for 10 min. One unit of GPOX activity was defined as the amount of enzyme that increased the absorbance by 0.01 per minute [38].

The total protein content in the supernatant was determined using the Bradford method. Briefly, 5 µL of the supernatant was mixed with 195 µL of Bradford reagent. After 5 min of incubation, the absorbance was measured at 595 nm. Protein content values were used to calculate the final specific enzyme activities [39].

### 3.4. Nutritional Status of American Cranberry Plants

The nutritional status of American cranberry plants was evaluated on the 30th day following planting stem cuttings. The collected plants were cleaned of substrate and then separated into underground and aboveground fractions. The aboveground parts were dried at 40 °C in a laboratory dryer with forced air circulation (POL-EKO-APARATURA, Włodzisław Śląski, Poland) until a constant weight was achieved. The samples were then homogenised using an IKA A11 Basic analytical mill (IKA Poland Sp. z o.o., Warsaw, Poland). The nitrogen (N) content in the plant material was determined by the Kjeldahl method according to the procedure described by Nelson et al. [35]. The contents of phosphorus (P), calcium (Ca), potassium (K), magnesium (Mg), iron (Fe), manganese (Mn), zinc (Zn), and copper (Cu) were analysed in plant material subjected to wet mineralisation using a mixture of concentrated mineral acids HNO_3_, HClO_4_, and H_2_SO_4_ in a ratio of 20:10:1 in an open system using a TECATOR heating block (FOSS, Hilleroed, Denmark). After mineralisation, the samples were transferred to volumetric flasks and diluted to a volume of 50 cm^3^ with deionised water. The phosphorus content was determined colorimetrically using the vanadomolybdate method according to the procedure described by Pége et al. [40]. The contents of the remaining elements were determined by atomic absorption spectrometry (AAS) using a HITACHI Z-2000 instrument (Hitachi, Tokyo, Japan).

### 3.5. Analysis of Selected Morphological Root Traits of Plants

To assess the effect of the developed coating preparations on root system development in American cranberry stem cuttings, the selected morphological root traits were measured. The analysed root traits included total root length, volume, surface area, and average diameter. Measurements were performed 30 days after planting. For this purpose, 50 plants were randomly sampled from each treatment group. To prepare the samples for root trait analysis, stem cuttings with newly developed root systems were first harvested by collecting 10 cm segments of the plants. The collected plant material was then carefully washed in a water bath to remove any remaining substrate while minimising the risk of damaging the delicate root structures. After cleaning, the selected morphological root traits were assessed using a scanning and image analysis system (WinRHIZO, Regent Instruments Inc., Quebec, QC, Canada), which allows for the precise quantitative analysis of root structure and development.

### 3.6. Analysis of Selected Chlorophyll Fluorescence Parameters

Measurements of the selected chlorophyll fluorescence parameters—maximum quantum efficiency of photosystem II (Fv/Fm) and maximum quantum efficiency of primary photochemistry of PSII (Fv/Fo)—in the leaves of American cranberry depending on the applied coating were conducted on the 30th day after transplanting stem cuttings. The measurements were carried out using a fluorimeter (Pocket PEA, Hansatech Instruments, King’s Lynn, Norfolk, UK) following the methodology described by Matlok et al. [41].

## 4. Conclusions

The application of auxin-enriched gel coatings, particularly the IBA W4 formulation, significantly improved root system development and physiological performance in large-fruited cranberry plants. These coatings contributed to optimised moisture conditions and reduced transpiration, thereby promoting more effective rooting and enhanced nutrient uptake. A well-developed root system was associated with elevated plant nutrient status and increased antioxidant enzyme activity, indicative of a balanced physiological state.

The demonstrated effectiveness of these coatings confirms their readiness for implementation in commercial nursery production. Their compatibility with mechanised planting systems has already been validated under practical conditions, confirming the scalability of the method. By improving rooting success and ensuring uniform plant development, these coatings offer a promising tool to increase propagation efficiency while reducing labour demands and resource inputs.

Furthermore, the use of sustained release gel matrices may contribute to greater environmental sustainability, as they minimise the need for repeated phytohormone applications and reduce potential leaching into the environment.

Although the method has proven effective under current conditions, future research should explore cost–benefit ratios, long-term field outcomes, and potential adaptation across other horticultural species, which would further expand the utility and impact of this propagation technology in modern horticulture.

## Data Availability

The original contributions presented in the study are included in the article, further inquiries can be directed to the corresponding author.
